# Some of the Things Which Make a Dentist Professional

**Published:** 1902-05-15

**Authors:** N. S. Hoff


### DENTAL REGISTER.

Vol. LVI. May 15, 1902. No. 5.

### SOME OF THE THINGS WHICH MAKE A DENTIST PROFESSIONAL.

BY N. S. HOFF, D.D.S.

Read before the Jackson and Southwestern Michigan Dental Associations, April 7, 1902.

As commonly defined a profession is an occupation that properly involves a liberal education or its equivalent, and whose practice requires mental rather than manual skill. The three professions often spoken of as the learned professions being those of religion, law and medicine. But other occupations of men are very properly classified professional when their pursuit or practice is based upon like or similar principles to those which characterize the three learned professions named. In modern athletics as soon as a man engages in athletic contests for pay he at once becomes and forever remains a professional ; and so it is with singers, actors, authors and others. But the mere fact of one having received pay for egaging in a public contest, or for writing a book, does not constitute him a professional in the highest and best sense. If it did, we fear that the innumerable army of ignorant, or at least unlettered people, who, by lurid public appeals of every conceivable kind, are ever endeavoring to attract public notice, by offering something of priceless worth for a small monetary compensation, would have to be classified as professors or professional. Indeed, we have already so stretched the significance of this term that we are accustomed to speak of the ignorant and wilfully idle tramp as a professional.

It is not however in this lower or popular significance that we want to define the office and functions of the learned and skillful practitioner of dentistry. We hope to call attention to his claims on the higher principles not only of education and skillful training, but because of the moral and beneficent influences which so largely predominate his calling.

It may not be necessary in the minds of any one here to even raise the question as to whether dentistry is a profession or not; because of the fact that it is historically and by virtue of its practice so closely allied with one of the three recognized professions. But dentistry is assuming such large proportions numerically, and its devotees receive their education and training so frequently entirely independent of medical schools, that it is coming to be thought of by the general public as a distinct calling. Without stopping here to discuss this question we will for our immediate purpose take the liberty of considering it as standing on its own basis and as a distinct occupation and not as a branch or specialty of the great healing art.

Since it is essential that all legitimate professions should be based upon the standard of a liberal education, let us see how strong the dentist's claim can be made for such a standard. It is probable that not many practitioners of to-day can lay claim to a liberal education on the basis of an academic degree ; there are many practitioners who have never even completed the course of study required to obtain the professional degree. Nevertheless, even they are serving the people so acceptably, and have by the virtue of their own energy and the demands of their calling, and the position accorded them in society, obtained a considerable amount of general and special knowledge and acquired such culture as was not known at the time when the three occupations of religion, law and medicine were first recognized as the learned professions. But we are not compelled to rest our case on the attainments of such representatives, much as we honor their attainments. This class of practitioners embraces a very small number comparatively.

It is well known that for a man to take up the practice of dentistry to-day, there is practically but one course open to him, namely: that he shall take the regular course of study in a dental college and when he has obtained a degree from such an organization he is practically accepted as qualified to take up the practice of his profession. It is true that some men enter practice without this course, but in practically every State in the Union, if a man can not present a diploma from a recognized reputable school stating that he has completed its curriculum of study satisfactorily to its faculty, he is not recognized as having any claim to offer his services to the public as a dentist. In some States an equivalent to the professional degree in the form of an examination by the State Board of Examiners may be accepted in lieu of a diploma. While this privilege may somewhat vitiate our evidence, because it is capable of being abused, it is a righteous provision when honestly enforced, and can not be greatly harmful as comparatively few are now entering our ranks in this manner. And this privilege is being withdrawn in one State after another until it will probably be but a short time until it will be removed from the laws of all States.

There are also a few men in the profession on credentials obtained from dental colleges in former times "*in absentia*," or without the curriculum, such degrees were conferred for meritorious contributions to the science or art of the profession, and were considered perfectly legitimate until this privilege was cut off by the action of the National Association of Dental Faculties about fifteen years ago, since which time no honor ary degrees have been granted by any American college. It is well known, however, that the greater number of these honorary degrees are in fact held by men of very high attainments professionally, in both the science and art of dentistry, and they are men also of recognized culture and worth in the communities where they live. Therefore it would seem that this comparatively small contingent should not in any way embarrass our claim as they undoubtedly measure up to the "equivalent" standard at least.

The vast majority of dental practitioners of to-day have been educated and trained in the professional schools specially equipped for this purpose, and a brief rehearsal of what this involves, will, we think, present a full and complete argument at least on the educational aspect of this subject.

The minimum entrance requirement for admission to our dental schools to-day is the completion of at least two year's of school-work in a graded high school, and some of the schools required a complete course of high school work, including two year's of Latin, or German and French, besides physics and elementary chemistry. In fact such preparation as admits to the better universities. It is true that this has not always been required, but a fair amount of literary training has been demanded for entrance to all dental schools for the past twenty-five years. And it is confidently hoped and expected that in the next five years all colleges will require a high school graduation or its equivalent for admission. If we add to this the three or four years of professional training, involving as it does, a careful study of the sciences of physics, chemistry, anatomy, bacteriology, and the numerous other kindred subjects which are embraced by the curriculum, can we not with confidence maintain our assumption of being not only professional, but by the standards of the commonly-accepted definition of being also a learned profession?

If then we have secured upon the educational basis a professional right, let us see if there may not be a similarly strong claim established on the basis of morality and beneficence. To develop this claim we shall have to enter as evidence that, which, while it may not be apparent to the general observer, is most real to every conscientious, thoughtful and experienced dental practitioner. It is to make good the claim that the highest ambition of the dentist is not, as may be supposed, self-aggrandizement; but an earnest purpose to serve his patrons to the best of his knowledge and ability, and with a fidelity that is not surpassed in any other calling. And we would say this, too, with all respect and reverence to the tender and pious ministry of the clergy, the wisdom and fidelity of the legal fraternity and the well-known devotion of the medical man to the poor and unfortunate. There is of course no such opportunity in a dentist's ordinary life, to encounter or develop the spectacular or heroic incidents more or less common to some other callings; but what we wish to maintain is that the morality and benevolence of his calling is the same in kind and quality as that which characterizes other professional callings, and that while it many times goes without even a word of quiet praise, is not the less kind and good in quality, and so entitled to a measure of credit not always accorded. To bring out this conception may we not best do so by briefly reviewing somewhat hurriedly the more prominent personal attributes of the ideal and successful practitioner as expressed in his habitual attitude toward his patients.

In order that a dentist may put himself in a position to acceptably serve such as may require his services, he feels called upon to suitably office or locate himself and have at his command such equipment in the way of technical facilities as the practice of his art in the most modern and humane manner may require. And just here we believe the difference between a truly professional man and the man who has mistaken his calling, or who conducts his practice entirely as a commercial enterprise, can be most clearly discerned. The professional man will look for a comfortable and respectable office and furnish it in a tasteful and harmonious manner, equipping it with every available aid which his means can command; but without offending the good sense of his patrons by a vulgar display of gaudy furniture and terrorizing machinery. That he should provide suitable and efficient means for hygienic cleanliness of person and instruments also shows a proper sense of his moral obligation. That dentists are now giving more thought and care to this particular department of their calling than ever before is one of the evidences of a quickened conscience and a determination to fulfill this obligation in a truly professional manner.

In the conduct of his practice there is great opportunity to distinguish the earnest and devoted practitioner from the charlatan. The intimate associations unavoidably required make integrity of moral character and actions absolutely essential. And it is with great satisfaction that our profession, considering the opportunities and provocations, has so little that is scandalous to reproach it in comparison with some others. It is one of the strongest evidences of the moral integrity which characterizes it.

It is however in the more positive virtues, that it seems to me, the dentist excells. There is no other practice of the healing art unless it be the practice of some branches of surgery where so much pain accompanies treatment as in dentistry. And this calls for continued and almost unlimited drafts on the nervous energy of the dentist, and also requires the most rigorous training in gentleness and kindness, that he may reduce to a minimum his necessarily painful task, and this, not that he may be the gainer, for all such efforts are a net loss of time and financial as well as nervous strength, but that the patient may not suffer more than is positively necessary. I am sure I need not further elaborate this statement to give it more force; but should you need a further illustration I need only cite the trying ordeal you are occasionally called upon to go through in a patient and gracious manner, while endeavoring to supply with artificial dentures the lost organs of mastication for a patient who seems to think her mission on earth, for the time at least, is to cultivate in you the virtues ot patience and perseverance. It is the overcoming of such difficulties and coming off victorious, not with harsh and cruel, or what we have sometimes heard called business-like methods; but, by painstaking effort, persistent kindness of manner and action, and a self-control which is akin to the divine, and which ought forever to stamp one as truly professional, that these difficulties are satisfactorily overcome. Such illustrations I might multiply at considerable length and add still more to the strength of the assertion of the moral and kindly attitude of the dentist, but there are other things which I wish at least to mention, and must therefore refrain from further comment, and I do so with a feeling that what I have tried here briefly to state can not be successfully controverted. My conviction is that the dentist is truly "long suffering and kind," and as a class his moral fibre is as closely and strongly knit as that of most other professional men even though they may make pretension of larger claims.

Is there anything in the private or social life of the dentist that is incongruous with his claims as a professional man? I have observed this phase of the dentist's life more or less thoughtfully for several years, and in my acquaintance with dentists have endeavored so far as I could to ascertain what domestic habits, social relations, participation in home and national politics, and interest in benevolent affairs has characterized them. I think you will believe me when I say that so far as I have observed it a very large proportion of dentists will be found actively interested in those things which go to make for the general welfare of the community in which they live. They are often leaders in politics, public enterprises, church and benevolent work. I have in mind a distinguished member of our profession who has been for twenty-five years the clerk or registrar of a religious conference. An honored practitioner of our own State took the time necessary out of his practice to serve his district in the State legislature for two terms, and with great credit to himself and to the welfare of the State. Incidentally I may add that while serving in this capacity he was able to have a law passed establishing the dental department at the State University, which has and will do so much in preparing men to practice dentistry intelligently and professionally. Such illustrations I know you can in your mind's eye, if not in your own consciousness, further augment, and I trust you will agree with me in the belief that they have an important value in making up the sum total of our claims to professional recognition on the basis of intelligent moral force and activity. It may be and often is said that men engage in politics or church work to extend their acquaintance for business reasons. But my experience has been that such careers are short lived. Men can not practice deceit for any considerable time without being found out, and by losing the public confidence lose their own self-respect and in due time fall into their rightful places. The demands of society are rigorous and imperative, and they are none the less exacting of him who attempts to practice dentistry honorably.

I wish to submit for your thought one more evidence which it seems to me should characterize us as professional. I submit it last because I believe it is the strongest evidence that we can offer. It is the spirit of progress, which we see most plainly manifested in our literature; supported in the main by the various local, State, national and international organizations; and in the remarkable advances made in the system and facilities for the education of dentists.

Fifty years ago there were not more than a half dozen dental societies, with five or six dental journals and but few text-books, and but two dental colleges in existence. At the present time there are over fifty dental colleges in the United States, involving more than a million dollars in property investment, more than thirty dental journals, and more dental societies than we can enumerate; there being eight or nine in this State alone. Other countries are similarly well equipped with these evidences of professional progress and development. The appearance of a new text-book or scientific treatise on some dental subject is now a frequent occurrence. All of which would seem to indicate that a spirit of learning has taken possession of us with a determination to place our technical practice on a scientific basis.

What significance has this evidence of a spirit of progress in literature and education in advancing the dentist's claim to be professional?

It may be possible that much of what seems to be progressive, in the activities referred to, is not seeking for knowledge from the highest motives, but results from a desire to satisfy personal ambitions for notoriety. We are sometimes reminded that colleges are run for the money or advertising there may be in them as business ventures; that books are the result of vanity and egotism; and that dental societies are run by cliques of men who desire an audience and place for airing their ideas. While it is possible that some or even most persons who engage actively in such works are at sometime in their careers influenced by a public consideration, we can not think that all the splendid achievements of the past in dentistry has sprung from any such low motives. In fact if we reflect on the men who have so largely contributed to this result we shall find that those by whom the most valuable advances have been brought about, have been men of singularly humble and devoted dispositions, and could not be accused of personal aggrandizement, because they have never sought nor attained it. The mercantile value of literary products on dental scientific subjects is so small that every one knows that but very few editors of books have received any adequate remuneration for their arduous labors, while most of them have lost money besides the valuable time and labor spent in producing them. A few business managers of dental colleges have probably received profitable remuneration for their time and money invested, but the large majority of college promoters and teachers would have been better off financially had they attended entirely to their practices. It is a fact that many men engage in teaching and spend time and money in attending dental society meetings, because of their love for the profession and the vitalizing influence of personal contact with men, who, like themselves, are striving to get the most out of their life's devotion to the calling which they are striving to elevate and dignify as a worthy occupation. We recently saw the statement in one of our dental periodicals that a Chicago dentist had attended twenty-one meetings of the New York Odontological Society. When we stop to consider what this involves; the traveling of nearly fifty thousand miles at an expense of ten thousand dollars for railway fares alone, to say nothing of hotel and other expenses and the inevitable loss of time out of his office, can we by any logic make ourselves believe that all this expense has been incurred to satisfy an unworthy ambition. We hardly think it possible. The truth of the matter is that the men who have done so much to develop the professional idea among dentists are actuated by unselfish motives, and it is only exceptional that we do find a man doing this sort of work from selfish motives. It is a truly professional spirit which seems to pervade the profession. Men who never engage in literary work themselves, or who are seldom seen at dental societies, are not less proud of our literature ; and abstain from active participation, not because they do not approve, but from modesty or habitual considerations. If there were time we could cite many illustrations proving that there is a true spirit of seeking after newer and better methods, with the hope that such discoveries may serve as equipment for more efficient service. We therefore maintain that the spirit of inquiry for truth, prosecuted successfully along scientific lines, entitles dentists to a professional classification for this reason if none other.

If then the dental education of to-day, and the almost universal habit of consecrating one's best energies to the welfare of his patrons, and the harmonious and concerted if not spontaneous efforts that are being made for the development of a higher class of dentists, and our accumulated literature do not entitle us to a professional recognition, we should cease to make any effort to increase our standards either educationally or morally. This would be calamatous, not only to our calling as a profession but also as a business.

There is scarcely another calling which demands more or which rests more securely on a basis of confidence or personal integrity. The dentist must not only be educated and skillful, but he must be thoroughly trustworthy. These qualities we believe characterize the great body of successful practitioners to-day, and entitle us to a professional recognition at least commensurate with our educational and moral attainments.

